# SARS-CoV-2 Resistance to Small Molecule Inhibitors

**DOI:** 10.1007/s40588-024-00229-6

**Published:** 2024-06-24

**Authors:** Uxua Modrego Lopez, Md. Mehedi Hasan, Brandon Havranek, Shahidul M. Islam

**Affiliations:** 1 Department of Chemistry, Delaware State University, Dover, DE 19901, USA; 2 Sidney Kimmel Medical College at Thomas Jefferson University, Philadelphia, PA 19107, USA

**Keywords:** SARS-CoV-2, 3CLpro, nsp12, small molecule therapeutics, Resistant mutations

## Abstract

**Purpose of the Review:**

SARS-CoV-2 undergoes genetic mutations like many other viruses. Some mutations lead to the emergence of new Variants of Concern (VOCs), affecting transmissibility, illness severity, and the effectiveness of antiviral drugs. Continuous monitoring and research are crucial to comprehend variant behavior and develop effective response strategies, including identifying mutations that may affect current drug therapies.

**Recent Findings:**

Antiviral therapies such as Nirmatrelvir and Ensitrelvir focus on inhibiting 3CLpro, whereas Remdesivir, Favipiravir, and Molnupiravir target nsp12, thereby reducing the viral load. However, the emergence of resistant mutations in 3CLpro and nsp12 could impact the efficiency of these small molecule drug therapeutics.

**Summary:**

This manuscript summarizes mutations in 3CLpro and nsp12, which could potentially reduce the efficacy of drugs. Additionally, it encapsulates recent advancements in small molecule antivirals targeting SARS-CoV-2 viral proteins, including their potential for developing resistance against emerging variants.

## Introduction

In March 2020, the World Health Organization declared COVID-19 as a global pandemic [1]. Since its emergence in December 2019 in Wuhan, China, there have been over 775 million reported cases of COVID-19 and approximately 7 million associated deaths [2]. The illness is attributed to severe acute respiratory syndrome coronavirus 2 (SARS-CoV-2).

SARS-CoV-2 is an enveloped, positive-sense, single-stranded RNA virus of the *Coronaviridae* family and has one of the largest genomes (~29.9 kb) among RNA viruses [3, 4]. The SARS-CoV-2 virus enters host cells primarily through a process involving the viral spike (S) protein and host cell receptors, ACE2 (angiotensin-converting enzyme 2) and TMPRSS2 (transmembrane protease serine 2). The spike protein binds to the ACE2 receptor and undergoes proteolytic cleavage by TMPRSS2, which leads to the activation of the spike protein and subsequent membrane fusion. After entering the host cells, viral RNA is translated into two large polyproteins, pp1a and pp1ab which are further processed by the main protease (M^pro^, also known as 3CLpro) and papainlike protease (PL_pro_) to generate 16 nonstructural proteins (nsps) [5, 6]. After 3CLpro mediated proteolytic processing, mature nsp12 coordinates with other non-structural proteins (nsp7 to nsp10, nsp13 to nsp16) in the viral replication-transcription complex (RTC) [6]. RTC catalyzes template unwinding, RNA synthesis, RNA proofreading, and RNA capping. The synthesized genomes are used for translation to generate more nsps and RTCs and are packaged into new virions. They are also translated to form different structural proteins (spike, envelope, membrane, nucleocapsid) and nine other accessory proteins, leading to the completion of assembly and release of new viral particles by exocytosis [7].

Various antiviral strategies have been developed. These include the design of mini-binders, peptides, ACE decoys, monoclonal antibodies, and small molecules, all aimed at blocking viral entry, neutralizing the virus, and inhibiting SARS-CoV-2 growth by targeting different stages of its life cycle [8–18]. Several antivirals targeting the spike protein (Bebtelovimab, Regdanvimab, etc.), 3CLpro (Paxlovid, Ensitrelvir), and nsp12 (Remdesivir, Molnupiravir, Favipiravir, etc.) got approval for emergency use in different countries [19, 20]. In contrast to antibody agents, small-molecule antivirals hold several advantages, including better pharmacokinetics, administration, cost-effectiveness, scalability in production, and ease of storage and transportation [21]. Currently, small-molecule antiviral drugs, Paxlovid (Ritonavir-Boosted Nirmatrelvir) and Remdesivir are FDA approved and Molnupiravir is authorized under FDA EUA (emergency use authorization) for COVID-19 treatment [22]. The use of Nirmatrelvir and Ritonavir for treating COVID-19 symptoms resulted in an 89% reduction in the risk of the disease progressing to severe stages [23]. Additionally, among non-hospitalized patients with high chance of COVID-19 progression, Remdesivir was also found to decrease the risk of hospitalization or death[24].

Due to the efforts by researchers and widespread vaccination campaigns, there has been notable improvement in the COVID-19 situation. However, it remains a significant concern due to the continuous mutation and evolution of SARS-CoV-2. Since its outbreak in 2019, SARS-CoV-2 has undergone over 500,000 mutations [25]. Certain mutations have resulted in the emergence of new Variants of Concern (VOCs), impacting transmissibility, illness severity, and the efficacy of antiviral medications. Notably, variants such as Alpha (B.1.1.7), Beta (B.1.351), Gamma (P.1), Delta (B.1.617.2), and Omicron (B.1.1.529) have been linked to heightened transmission rates and reported reductions in the effectiveness of antiviral treatments and vaccines. [26]. Additionally, Omicron variants XBB.1.5 and BA.2.86 are currently Variants of Interest (VOIs) [27]. As of April 30, 2024, the Omicron variant JN.1 is predominant in the United States [28]. Monoclonal antibodies like Bebtelovimab, Regdanvimab, and Sotrovimab, previously used for COVID-19 treatment, are now ineffective against newer variants of Omicron such as BQ and XBB. Resistance has also been observed with combination therapies such as REGEN-COV, Evusheld, Bamlanivimab, and Etesevimab [19]. This highlights the urgency for new therapeutics as current drugs may not be effective against future VOCs, echoing past challenges seen in other viruses like hepatitis B, HIV-1, HCV, and influenza [29–32].

The precise monitoring of drug resistance mutations in COVID-19 treatment is crucial for alerting individuals to potential reductions in drug effectiveness due to mutations. Furthermore, the identification of drug-resistant mutations is essential for the development of newer, more potent drugs with reduced susceptibility to viral resistance. In this context, here we summarize potential resistant mutations in five small-molecule drugs—Nirmatrelvir, Ensitrelvir, Remdesivir, Favipiravir, and Molnupiravir—currently approved and utilized for COVID-19 treatment in various countries, based on recent research findings.

## Discussion

**Fig. 1:**
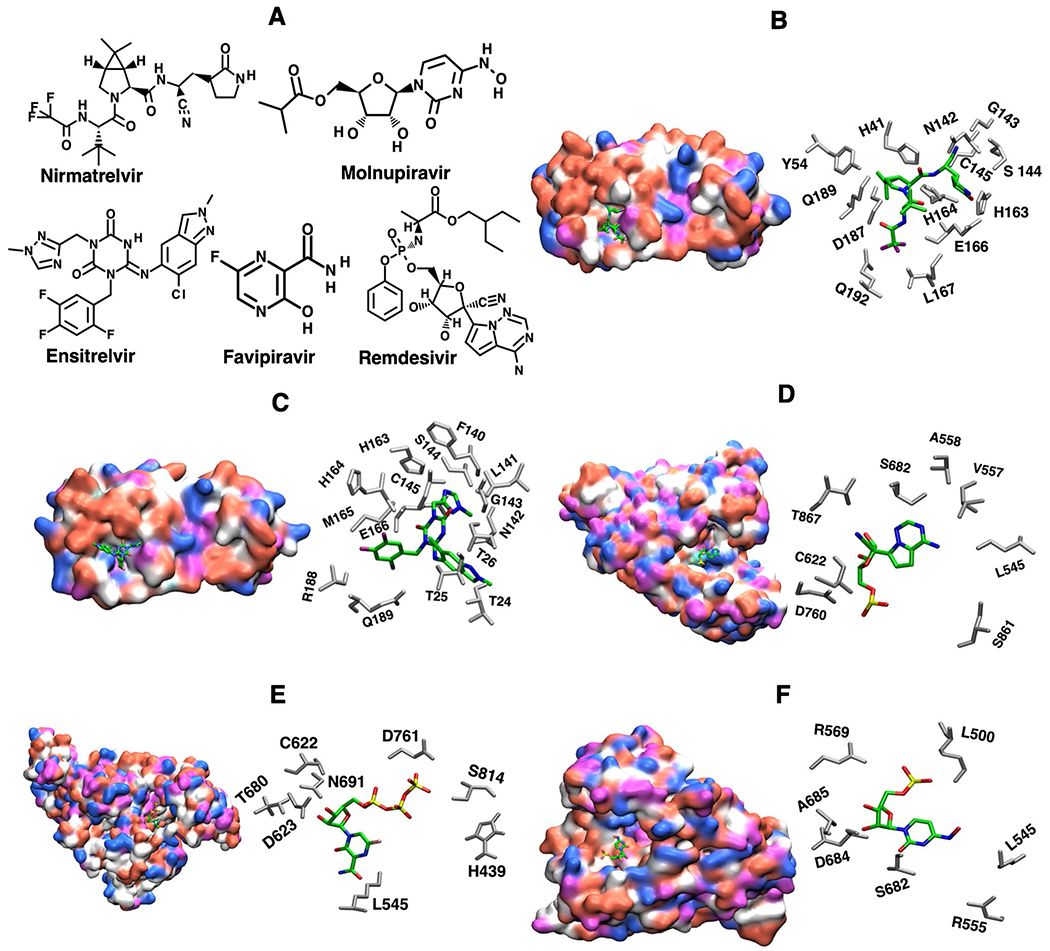
**A**) Chemical structures of small molecules approved for the treatment of SARS-CoV-2, Nirmatrelvir, Molnupiravir, Ensitrelvir, Favipiravir, and Remdesivir; **B**) Nirmatrelvir bound with 3CLpro (PDB ID 7VH8), **C**) Ensitrelvir bound with 3CLpro (PDB ID 8DZ0); **D**) Remdesivir bound with nsp12 (PDB ID 7BV2 ); E) Favipiravir bound with nsp12 (PDB ID 7AAP) and F) Molnupiravir bound with nsp12 (PDB ID 7OZU). Important residues that interact with these small molecule drug therapeutics are also displayed. Acidic, basic, polar, and non-polar residues are shown in magenta, blue, red, and white, respectively

Small molecule therapeutic drugs such as Nirmatrelvir, Ensitrelvir, Remdesivir, Favipiravir, and Molnupiravir have been approved for the treatment of COVID-19 (Fig. 1). These antiviral drugs either bind to 3CLpro or nsp12 proteins, inhibiting their function and leading to reduced viral replication. However, SARS-CoV-2 is continuously mutating and the potential for drug resistance has been made clear through experimental studies.

### Approved Antiviral Drugs Targeting 3CLpro

3-chymotrypsin-like protease (3CLpro), also known as main protease (M^pro^), is a cysteine protease containing three domains. Domain I (residues 8 -101) includes the catalytic dyad composed of residues His41 and Cys145. Domain II (residues 102-184) and domain III (residues 201-303) mainly help in dimerization and give stability to the enzyme structure [29, 30]. The catalytic dyad, located within domain I, is crucial for the enzymatic activity of the 3CLpro. His41 acts as a general base, abstracting a proton from the thiol group of Cys145, which then performs a nucleophilic attack on the carbonyl carbon of the peptide bond in the substrate, leading to proteolysis [31]. 3CLpro cuts the polyprotein at 11 conserved sites, starting with the autolytic cleavage of this enzyme itself from pp1a and pp1ab [32]. 3CLpro exclusively cleaves polypeptides after a glutamine (Gln) residue, and no known human protease displays the same cleavage specificity as 3CLpro [33, 34]. Therefore, the 3CLpro emerged as an ideal target for developing antiviral drugs. Hundreds of inhibitors have been developed targeting 3CLpro [19]. Presently, Nirmatrelvir (Paxlovid) holds FDA approval and is extensively employed in COVID-19 treatment, whereas Ensitrelvir has been approved and utilized in Japan since 2023 [22, 35]. The emergence of drug resistance mutations in 3CLpro associated with Nirmatrelvir and Ensitrelvir could pose a threat to COVID-19 patients. Table 1 represents mutations in 3CLpro that have been associated with drug resistance.

### Reported Nirmatrelvir Resistant Mutations in SARS-CoV-2

Pfizer’s oral drug Paxlovid containing Nirmatrelvir (PF-07321332) is a peptidomimetic covalent inhibitor of 3CLpro. Zhao et al. reported the crystal structure of SARS-CoV-2 3CLpro in complex with Nirmatrelvir (PDB 7VH8) with a resolution of 1.59Å, which showed residues H41, M49, Y54, F140, L141, N142, S144, C145, H163, H164, M165, E166, L167, H172, D187, R188, Q189, T190, and Q192 are interacting with Nirmatrelvir [45] . Nirmatrelvir forms a covalent bond with the Sγ atom of the C145 residue of 3CLpro. The imine nitrogen of the thioimidate group takes place in the oxyanion hole, and the backbone NH of G143 and C145 stabilize it through two hydrogen bonds. The oxygen and nitrogen atoms of the lactam ring interact with residues H163 and E166 by forming hydrogen bonds which also stabilize the inhibitor. Moreover, the amide nitrogen of Nirmatrelvir forms another hydrogen bond with the carbonyl oxygen of H164 [45]. Therefore, alteration in these critical residues could compromise Nirmatrelvir binding and develop resistance.

Residues H41, C145, and H163 are necessary for 3CLpro enzymatic activity and are improbable to mutate naturally to provide resistance against Nirmatrelvir [37•]. Havranek et al., employed alchemical free energy perturbation (FEP) alanine scanning to recognize potential resistance mutations in 3CLpro, focusing on residues within 4 Å of Nirmatrelvir, while excluding those essential for activity [36•]. The value of the free energy difference (ΔΔ*G*_FEP_) between the bound state (3CLpro-Nirmatrelvir) and free state (3CLpro) was calculated, where positive ΔΔ*G*_FEP_ value indicates unfavorable for binding. Y54A, S144A, and E166A were found to have positive ΔΔ*G*_FEP_ values, 2.96 kcal/mol, 2.92 kcal/mol, and 1.30 kcal/mol, respectively, suggesting these mutations could be detrimental to the effectiveness of Paxlovid. To validate these computational findings, IC_50_ of Nirmatrelvir was determined for the mutation combinations, Y54A/S144A and S144A/E166A. The IC_50_ values were found to be 0.400 ± 0.100 μM and 3.60 ± 0.64μM for Y54A/S144A and S144A/E166A, respectively and 0.051 ± 0.0064μM for the wildtype [36•].

In a separate computational and in-vitro study, Sasi et al., demonstrated the mutations N142L, E166M, Q189E, Q189I, and Q192T are important for 3CLpro function, but these mutations significantly decrease the Nirmatrelvir activity. N142L, E166M, Q189E, Q189I and Q192T have increased the IC_50_ values by about 9-fold, 24-fold, 19-fold, 4-fold, and 17-fold, respectively [38•]. Though the frequency of these resistant mutations was observed to be low according to the GISAID database, the emergence of these mutations could make them highly resistant to Nirmatrelvir [46]. Hu et al., highlighted residues S144, M165, E166, H172, and Q192 as hot spots for drug resistance as multiple mutations are found to these residues to become resistant to Nirmatrelvir. Twenty mutations (S144M/F/A/Y/G, M165T, E166G, H172Q/F, and Q192T/S/L/A/I/P/H/V/W/C/F) were identified in patients using GISAID sequences and observed to cause significant resistance to Nirmatrelvir (K_i_ increase by more than 10-fold), while maintaining enzymatic activity (K_cat_/k_m_ values within 10-fold variation to wildtype) [37•]. Moghadasi et al. acknowledged that natural 3CLpro variation consisting of the deletion of residue P168 (ΔP168) resulted in significant resistance to Nirmatrelvir when combined with other mutations such as A173V, D48Y, M49I, and M49L (IC_50_ increments compared to wild-type; ΔP168/A173V ~51-fold, D48/ΔP168 ~46-fold, M49I/ΔP168 ~8-fold, and M49/ΔP168 ~7-fold) [41••]. Additionally, in-vitro results from a study conducted by Jochmans et al. showed that mutations L50F, E166A, and L167F combined conferred up to ~80-fold resistance. In this combination, E166A and L167F were identified as being responsible for resistance while L50F is a compensatory substitution in charge of restoring fitness [39]. Double mutants M49L/S144A, L50F/E166A, L50F/E166V, and ΔP168/A173V also result in a major increase in the IC_50_ values of Nirmatrelvir (M49L/S144A ~11-fold, L50F/E166A ~27-fold, L50F/E166V >300-fold, and ΔP168/A173V ~55-fold, Table 1) [40••]. Hence, the appearance of more than one mutation simultaneously could result in a higher level of resistance than single mutations.

### Mutations Identified to be Resistant Against Ensitrelvir

Ensitrelvir fumaric acid, S-217622, is a nonpeptidic, noncovalent SARS-CoV-2 3CLpro inhibitor [47]. Lin et al. identified several residues of 3CLpro, important for Ensitrelvir binding (Fig. 1C) [48]. The sidechain imidazole group of H163 forms a hydrogen bond with the 1-methyl-1H-1,2,4- triazole group of Ensitrelvir and the side chain of H41 forms a π-π stack with 2,4,5-trifluoromethyl group. In addition, residues H163, C145, G143, and Q189 are involved in the hydrogen-bond network to stabilize the binding of Ensitrelvir. Residue E166 and F140 form 4 hydrogen bonds with S1 and G2 from the other promoter of the dimer to stabilize the binding pocket [48]. Therefore, any mutations in these residues or residues close to the binding region are expected to be detrimental to Ensitrelvir binding.

Moghadasi et al. studied the resistance of 3CLpro inhibitors in vitro and found that single mutations M49I/L, S144A, E166A/V, L167F, and deletion of P168 (ΔP168) increase the IC_50_ values of Ensitrelvir (about 5-fold to 75-fold, Table 1) [40••]. Variants with double mutations, M49L/S144A, L50F/E166A, and L50F/E166V, cause resistance to Ensitrelvir (high IC_50_ fold changes relative to wildtype, between ~21-fold and ~170-fold increases) [40••]. In a different study, it was found that single mutations (T45I, D48Y, M49I/L/T/V, S144A and ΔP168) and double mutants (T45I/M49L, T45I/A173V, ΔP168/A173V, D48Y/ΔP168, M49I/ΔP168, and M49L/ΔP168) conferred resistance to Ensitrelvir (IC_50_ increments between ~3 and ~127-fold change) [41••]. M49L, E166A, and M49L/E166A mutations also maintained viral fitness in vitro and in vivo, which could potentially be a threat to its effectiveness if expressed in the population (IC_50_ increases with respect to the wild-type ~61-fold, ~9-fold, and 197-fold, respectively) [43•]. Iketani et al., tested several single point mutants (T21I, L50F, S144A, E166V, A173V, P252L, T304I) and multiple mutants (T21I/S144A, T21I/E166V, L50F/E166V T21I/A173V, T21I/T304I, and T21I/A173V/T304I) for resistance to Ensitrelvir. Most of the mutations, except the T21I/A173V, T21I/T304I, and T21I/A173V/T304I, were found to be resistant to Ensitrelvir. All these mutations showed resistance to Nirmatrelvir (Table 1) [42•].

Overall, 39 single mutations in 17 critical 3CLpro residues and 19 combined mutants were identified to be resistant to Nirmatrelvir, Ensitrelvir, or both (Table 1). Mutations T21I, L50F, S144A, E166A, E166V, L167F, ΔP168, A173V, P252L, and T304I are of special interest as they showed greater resistance (higher IC_50_) compared to other mutations. Additionally, nine double mutants (T21I/S144A, T21I/E166V, M49L/S144A, L50F/E166A, L50F/E166V, ΜP168/A173V, D48Y/ΜP168, M49I/ΔP168, M49L/ΔP168, and T45I/A173V) and a triple mutant (L50F/E166A/L167F) were observed to be resistant to both 3CLpro inhibitors [39, 42•] . These mutants are critical as they confer resistance to both Nirmatrelvir and Ensitrelvir inhibitors which increase the probability of treatment failure. Continuous monitoring of these mutations is necessary to improve antiviral treatments and avoid 3CLpro inhibitor resistance.

### Antiviral Drugs Targeting Non-Structural Protein 12 (nsp12)

RNA dependent RNA polymerase (RdRp) is an essential enzyme consisting of a non-structural protein 12 (nsp12) core catalytic unit, a non-structural protein 7-non-structural protein 8 (nsp7-nsp8) heterodimer, and an additional nsp8 subunit [49]. The nsp12 or RdRp is a prominent target for small molecules as it is responsible for the replication of structural protein RNA. Its sequence is conserved across different coronaviruses, with about 96% similarity between SARS-CoV-2 and SARS-CoV [50]. Nsp12 is composed of an N-terminal nidovirus RdRp-associated nucleotidyltransferase domain (NiRAN), a dynamic interface domain, and a C-terminal RdRp domain. NiRAN domains function for RNA capping and the C-terminal RdRp domain conducts viral RNA synthesis [51, 52]. During viral RNA capping, the NiRAN domain adds a guanosine 5′-triphosphate to the 5′-end of viral RNA [53]. Several nucleotide analogs like Remdesivir, Molnupiravir, and Favipiravir can inhibit nsp12 function by chain termination or lethal mutagenesis [54–56]. Table 2 represents mutations in nsp12 that could be associated with drug resistance.

### SARS-CoV-2 Mutations Resistant to Remdesivir

Remdesivir (RDV), GS441524, is an adenosine nucleoside analog that inhibits RNA synthesis by targeting SARS-CoV-2 RNA-dependent RNA polymerase, nsp12 [63, 64]. After administration in the body, RDV is metabolized into RDV triphosphate [65] where it inhibits nsp12 through two different mechanisms: delay chain termination and template-dependent inhibition. In the first mechanism, RDV triphosphate is incorporated in the growing RNA which leads to the formation of a translocation barrier that causes the process to stall at position i + 3, i.e. three nucleotides down-stream of the site where RDV was incorporated [63, 66]. High concentrations of ribonucleoside triphosphate (NTP) overcome delayed chain termination resulting in full-length products where RDV triphosphate is retained in the RNA template. Delayed chain termination is linked with a stearic clash caused by the interaction of nsp12 residue S861 and incorporated RDV. Therefore, mutations of S861 could disrupt interaction with RDV resulting in potential inhibition of delayed chain mechanism. A second mechanism takes place in high concentrations of NTP when RDV triphosphate is retained in the RNA template; a stearic problem caused by nsp12 residue V557 arises when uridine triphosphate (UTP) is incorporated opposite to RDV in the template leading to inhibition [61].

Several SARS-CoV-2 mutation cases have been reported in patients undergoing RDV treatment. Mutation E802D in nsp12 was found in a 70-year-old immunocompromised patient after 7 days of RDV treatment. Gandhi et al. studied E802D in-vitro and found a significantly higher IC_50_ value than wildtype (4.2 μM vs 0.7 μM). They also studied the E802A mutation and found a 4 times increment in IC_50_ values compared to wildtype (2.7 μM vs 0.7 μM, Table 2) [57•]. These findings confirm the importance of residue E802 in RDV resistance. Hedskog et al. analyzed viral resistance from trial ACTT-1 and observed that two patients developed V791I and C799F mutations in nsp12 associated with 2.2 to 2.5-fold lower RDV susceptibility (EC_50_, 0.50 μM and 0.58 μM respectively vs 0.24 μM of wildtype, Table 2) [58•]. Mutations in nsp12 residue S861 could cause inhibition of the delayed chain terminator mechanism as this residue causes a steric clash when interacting with RDV. Tchesnokov et al. identified that the small residue mutation S861G shows no delayed chain termination and results in the production of full-length products while residue mutation S861P increases UTP concentration overcoming delated chain termination [61]. Similarly, mutation S861A was observed to reduce chain termination effectiveness [60]. Nsp12 mutations like P323L and D848Y and some other mutations in different non-structural proteins such as A504V in nsp14, and I115L in nsp15 were also observed in different stages of the treatment period, but resistance to RDV has not yet been confirmed [57•, 67, 68].

The emergence of RDV-resistant mutations requires high passage numbers and results in a low impact on susceptibility and high fitness cost [69]. Furthermore, Hedskog et al., showed a similar rate of nsp12 substitution emerging in patients under RDV treatment and placebo patients [58•]. This observation indicates that mutations could emerge because of natural viral evolution rather than RDV itself.

### In-silico Prediction of SARS-CoV-2 Mutations Resistant to Favipiravir

Favipiravir (6-fluoro-3-oxo-3,4-dihydropyrazine-2-carboxamide) is a purine base analog used as an antiviral drug against RNA viruses, and it has also shown effectiveness against SARS-CoV-2 [70, 71]. This treatment can reduce the viral load leading to faster clinical improvements [72]. Favipiravir has been used in different countries around the world including Japan, Egypt, Russia, and China [1, 72]. There are two different mechanisms of action of Favipiravir against SARS-CoV-2. It acts as a chain terminator or attacks nsp12 through mutagenesis [73]. Once Favipiravir is introduced into the body, it is activated and converted into Favipiravir ribofuranosyl 5’-triphosphate (Favipiravir RTP) through ribosylation and phosphorylation. It is incorporated into the active site of RdRp where it is mistaken for a purine-causing chain termination [74]. Favipiravir can also act as a mutator by inducing C to A and G to A mutations when inserted into viral RNA causing lethal mutagenesis in the SARS-CoV-2 genome [75].

Favipiravir RTP interacts with SARS-CoV-2 at the catalytic site of RdRp. This interaction is coordinated by nsp12 residue L545 which forms hydrogen bonds with the nitrogen atom in the pyrazine ring or donates the fluorine atom of Favipiravir RTP. Nsp12 residue N691 also forms a hydrogen bond with the 2’hydroxyl of Favipiravir RTP [76]. The role of L545 and N691 is critical as a mutation in these residues could disrupt hydrogen binding decreasing the susceptibility to Favipiravir. Padhi et al., applied the resistance mutation scan methodology employed in a Molecular Operating Environment (MOE) to identify potential mutations [59•]. A total of 350 mutant versions of the Favipiravir-nsp12 complex were generated and the relative binding affinity ΔAffinity), which represents the Boltzmann average of the relative affinities of the new complexes was calculated. H439D, H439L, C622R, C622F, C622Y, D623A, D623G, D623V, T680A, T680K, T680M, T680P, and T680S a total of 13 mutations were found to have ΔAffinity values greater than 0.2 kcal/mol indicating potential resistance to Favipiravir. Among them, three mutations, C622R, T680K, and T680S, were observed to have the highest ΔAffinity values (ΔAffinity > 0.4 kcal/mol) demonstrating valuable potential Favipiravir resistance if present in nsp12. Additionally, D623A and D623G exhibit special interest as they are predicted to be Favipiravir resistant by both MOE-based resistance design and Rosetta-based design method [59•]. Although SARS-CoV-2 resistance to Favipiravir has not been observed clinically yet, several in-vitro experiments found RdRp-resistant mutations to Favipiravir for other viruses such as Chikungunya virus and Enterovirus [77–79].

### Mutations Observed in SARS-CoV-2 During Treatment with Molnupiravir

Molnupiravir is an antiviral drug used for the treatment of COVID-19 and other diseases caused by RNA viruses [80]. It has shown effectiveness against different strains of the virus such as alpha, beta, and gamma [81]. The MOVeOUT trial reported successful results in unvaccinated patients with early COVID-19 reducing viral load, hospitalization, and mortality [82]. The primary results of the PANORAMIC trial did not confirm the effectiveness of Molnupiravir, but secondary outcomes showed promising results indicating faster recoveries in patients under Molnupiravir treatment [83]. It is administered orally and rapidly metabolized into the ribonucleoside β-D-N4-hydroxycytidine (NHC). NHC is then phosphorylated into NHC triphosphate (MPT) which binds to nsp12 and acts as an analog of cytosine and uracil, causing transition mutations from G to A and C to U. Accumulation of these errors in the viral genome results in lethal mutagenesis [84]. NHC interacts with the RdRp active site constructed by nsp12 residues L545 to R555. More specifically, L545 forms a hydrogen bond with the nitrogen atom in the pyrimidine ring while R555 forms hydrogen bonds with an oxygen atom in NHC [85]. Mutation A716V has also been noted in nsp12 among patients undergoing treatment with Molnupiravir, but its resistance to Molnupiravir has not been verified [86•]. Based on the findings so far, the impacts of the mutations observed in Molnupiravir treatment and in vitro experiments are unknown. However, they must be monitored since they can be transmitted from patient to patient when the viruses are not eliminated from the host under treatment.

### Mutations may Arise in SARS-CoV-2 Spike Protein due to Treatments with Antiviral Drugs

The spike protein of SARS-CoV-2 is vital for infecting host cells and is a key target for antibodies and vaccines. Mutations in this protein can affect how easily the virus spreads, its ability to evade the immune system and the effectiveness of vaccines. Although there are no reports of mutations in spike protein for patients receiving Nirmatrelvir, some patients receiving Remdesivir have shown amino acid, 141–144 or 141–145, deletions in the spike protein [87, 88]. Studies have also reported various mutations in the spike protein among patients treated with Molnupiravir, including the F460S mutation, which may affect its binding to the ACE2 receptor [86•]. Extending the treatment duration, utilizing combinations of antivirals, and redesigning the drugs could be effective strategies to reduce the risk of the emergence of new SARS-CoV-2 variants and to lower transmission and reinfection rates.

## Conclusion

Several small-molecule drugs such as Remdesivir, Ensitrelvir, Favipiravir, Molnupiravir, and Nirmatrelvir (Paxlovid), are currently in use for COVID-19 management. The drugs mainly target two important viral proteins, 3CLpro and RdRp, to inhibit viral growth. A higher mutation rate is a distinctive characteristic of RNA viruses like SARS-CoV-2. Mutations can impact the efficacy of antiviral treatments, necessitating vigilant monitoring to prevent treatment ineffectiveness. SARS-CoV-2 variants have already become resistant to several monoclonal antibodies used for COVID-19 treatment.

Recent studies have identified several mutations associated with resistance to Nirmatrelvir and Ensitrelvir in 3CLpro. There are 17 critical residues located in 3CLpro where a total of 39 different mutations have been observed to reduce the inhibitory activity of Nirmatrelvir and Ensitrelvir. Mutations in E166 have been found to be recurrent and showed high IC_50_ values in several studies. 3CLpro single mutations (T21I, L50F, S144A, E166A, E166V, L167F, A173V, P525L, and T304I) and deletion of P168 can raise resistance to both drugs, which can trigger treatment failure. Furthermore, the appearance of more than one 3CLpro mutation simultaneously could increase IC_50_ values by multiple times more than single mutations as shown in the L50F/E166V double mutant. Mutations in nsp12 were found to be comparatively less frequent than mutations in 3CLpro. Single mutations like E802A, E802D, V791I, and C799F in nsp12 have shown Remdesivir resistance. Recent findings indicate that resistance to Favipiravir could potentially emerge with H139, C622, D623, and T860 residues in nsp12. Further studies are required to identify Molnupiravir resistance mutations. Patients with some antiviral treatments have been shown to develop mutations in the spike proteins which could lead to improved transmissibility of the virus and can promote re-infection. Drug resistance poses a significant challenge necessitating vigilant monitoring, given its potential for the emergence of resistant variants of SARS-CoV-2. Hence, sustained investigation is imperative to detect novel resistant mutations, thereby safeguarding the efficacy of current therapeutics and advancing insights for the development of more efficacious treatment modalities.

## Figures and Tables

**Table 1:** Reported mutations in 3CLpro that are associated with resistance to Nirmatrelvir and Ensitrelvir and the corresponding IC_50_ values and their fold increase due to mutation

SARS-CoV-2 3CLpro variant	Antiviral Small-Molecule	IC_50_[nM]^a,b^	Increment of IC_50_^b^	Reference**
Wildtype	Nirmatrelvir	51		[36•]
	Nirmatrelvir	26		[37•]
	Nirmatrelvir	9		[38•]
	Nirmatrelvir	23		[39]
	Nirmatrelvir	29		[40••]
	Nirmatrelvir	32		[41••]
	Nirmatrelvir	51		[42•]
	Ensitrelvir	25		[39]
	Ensitrelvir	36		[40••]
	Ensitrelvir	23		[41••]
	Ensitrelvir	190		[43•]
	Ensitrelvir	13		[42•]
T21I	Nirmatrelvir	233	~5-fold	[42•]
	Ensitrelvir	21	~2-fold	[42•]
T45I	Ensitrelvir	111	~4-fold	[41••]
D48Y	Ensitrelvir	135	~5-fold	[41••]
M49I	Ensitrelvir	335	~12-fold	[41••]
	Ensitrelvir	338	~9-fold	[40••]
M49L	Ensitrelvir	436	~25-fold	[41••]
	Ensitrelvir	769	~21-fold	[40••]
	Ensitrelvir	11,600	~61-fold	[43•]
M49T	Ensitrelvir	69	~4-fold	[41••]
M49V	Ensitrelvir	45	~3-fold	[41••]
L50F	Nirmatrelvir	215	~4-fold	[42•]
	Ensitrelvir	34	~3-fold	[42•]
N142L	Nirmatrelvir	85	~9-fold	[38•]
S144A	Nirmatrelvir	171	~7-fold	[37•]
	Nirmatrelvir	236	~8-fold	[40••]
	Nirmatrelvir	267	~12-fold	[41••]
	Nirmatrelvir	24	~2-fold	[42•]
	Ensitrelvir	623	~17-fold	[40••]
	Ensitrelvir	166	~13-fold	[42•]
	Ensitrelvir	395	~17-fold	[41••]
S144M	Nirmatrelvir	175	~7-fold	[37•]
S144F	Nirmatrelvir	133	~5-fold	[37•]
S144Y	Nirmatrelvir	61	~2-fold	[37•]
S144G	Nirmatrelvir	97	~4-fold	[37•]
M165T	Nirmatrelvir	95	~4-fold	[37•]
E166A	Nirmatrelvir	230	~10-fold	[39]
	Nirmatrelvir	622	~21-fold	[40••]
	Ensitrelvir	126	~35-fold	[40••]
	Ensitrelvir	1,720	~9-fold	[43•]
E166M	Nirmatrelvir	218	~24-fold	[38•]
E166G	Nirmatrelvir	92	~ 4-fold	[37•]
E166V	Nirmatrelvir	>10,000	>300-fold	[40••]
	Nirmatrelvir	5,100	~100-fold	[42•]
	Ensitrelvir	2,800	~78-fold	[40••]
	Ensitrelvir	294	~23-fold	[42•]
L167F	Nirmatrelvir	100	~4-fold	[39]
	Nirmatrelvir	282	~10-fold	[40••]
	Ensitrelvir	728	~20-fold	[40••]
ΔP168*	Nirmatrelvir	243	~8-fold	[40••]
	Nirmatrelvir	180	~5-fold	[41••]
	Ensitrelvir	193	~5-fold	[40••]
	Ensitrelvir	157	~7-fold	[41••]
H172Q	Nirmatrelvir	152	~6-fold	[37•]
H172F	Nirmatrelvir	212	~8-fold	[37•]
A173V	Nirmatrelvir	460	~16-fold	[40••]
	Nirmatrelvir	328	~12-fold	[41••]
	Nirmatrelvir	88	~2-fold	[42•]
	Ensitrelvir	22	~2-fold	[42•]
Q189E	Nirmatrelvir	173	~19-fold	[38•]
Q189I	Nirmatrelvir	38	~4-fold	[38•]
Q192T	Nirmatrelvir	103	~4-fold	[37•]
	Nirmatrelvir	151	~17-fold	[38•]
Q192S	Nirmatrelvir	217	~8-fold	[37•]
Q192L	Nirmatrelvir	122	~5-fold	[37•]
Q192A	Nirmatrelvir	141	~5-fold	[37•]
Q192I	Nirmatrelvir	110	~4-fold	[37•]
Q192P	Nirmatrelvir	135	~5-fold	[37•]
Q192H	Nirmatrelvir	169	~7-fold	[37•]
Q192V	Nirmatrelvir	96	~4-fold	[37•]
Q192C	Nirmatrelvir	97	~4-fold	[37•]
Q192F	Nirmatrelvir	93	~4-fold	[37•]
Q192W	Nirmatrelvir	66	~3-fold	[37•]
P252L	Nirmatrelvir	297	~6-fold	[42•]
	Ensitrelvir	23	~2-fold	[42•]
T304I	Nirmatrelvir	278	~6-fold	[42•]
	Ensitrelvir	19	~2-fold	[42•]
T21I/S144A	Nirmatrelvir	478	~9.4-fold	[42•]
	Ensitrelvir	231	~18-fold	[42•]
T21I/E166V	Nirmatrelvir	4,230	~83-fold	[42•]
	Ensitrelvir	42	~3-fold	[42•]
T21I/A173V	Nirmatrelvir	160	~3-fold	[42•]
T21I/T304I	Nirmatrelvir	168	~3-fold	[42•]
T21I/A173V/T304I	Nirmatrelvir	756	~15-fold	[42•]
Y54A/S144A	Nirmatrelvir	400	~8-fold	[36•]
S144A/E166A	Nirmatrelvir	3600	~71-fold	[36•]
L50F/E166A/ L167F	Nirmatrelvir	1,600	~72-fold	[39]
	Ensitrelvir	2,300	~93-fold	[39]
M49L/S144A	Nirmatrelvir	321	~11-fold	[40••]
	Ensitrelvir	6,110	~170-fold	[40••]
M49L/E166A	Ensitrelvir	37,400	~197-fold	[43•]
L50F/E166A	Nirmatrelvir	793	~27-fold	[40••]
	Nirmatrelvir	2,700	~53-fold	[42•]
	Ensitrelvir	1,040	~29-fold	[40••]
	Ensitrelvir	46	~4-fold	[42•]
L50F/E166V	Nirmatrelvir	>10,000	>300-fold	[40••]
	Ensitrelvir	751	~21-fold	[40••]
ΔP168/A173V	Nirmatrelvir	1510	~51-fold	[41••]
	Nirmatrelvir	1630	~55-fold	[40••]
	Ensitrelvir	91	~3-fold	[41••]
D48Y/ΔP168	Nirmatrelvir	921	~46-fold	[41••]
	Ensitrelvir	754	~40-fold	[41••]
M49I/ΔP168	Nirmatrelvir	151	~8-fold	[41••]
	Ensitrelvir	990	~53-fold	[41••]
M49L/ΔP168	Nirmatrelvir	144	~7-fold	[41••]
	Ensitrelvir	2400	~127-fold	[41••]
T45I/M49L	Ensitrelvir	1030	~55-fold	[41••]
T45I/A173V	Nirmatrelvir	414	~21-fold	[41••]
	Ensitrelvir	79	~4-fold	[41••]

a IC_50_ (half-maximal inhibitory concentration), indicates how much drug is necessary to inhibit the action of the process by half [44]

b IC_50_ values and fold increase are rounded to the nearest whole number

* ΔP168 indicates deletion of Proline residue at position 168

** Various assays may be employed to obtain the findings; please refer to the respective publications for more information

**Table 2:** Reported mutations in nsp12 that are associated with resistance to Remdesivir and Favipiravir and the corresponding IC_50_, EC_50_ and ΔAffinity due to mutation

SARS-CoV-2 nsp12 variant	Antiviral Small Molecules	IC_50_ [nM]^a^, EC_50_ [nM]^b^ and ΔAffinity (kcal/mol)^c^	Reference**
Wildtype	Remdesivir	700^a^	[57•]
	Remdesivir	240^b^	[58•]
H439D	Favipiravir	0.2^c^	[59•]
H439L	Favipiravir	>0.2^c^	[59•]
C622R	Favipiravir	>0.5^c^	[59•]
C622F	Favipiravir	>0.2^c^	[59•]
C622Y	Favipiravir	>0.3^c^	[59•]
D623A	Favipiravir	~0.3^c^	[59•]
D623G	Favipiravir	>0.2^c^	[59•]
D623V	Favipiravir	>0.2^c^	[59•]
T680A	Favipiravir	~0.4^c^	[59•]
T680K	Favipiravir	>0.4^c^	[59•]
T680M	Favipiravir	~0.3^c^	[59•]
T680P	Favipiravir	~0.3^c^	[59•]
T680S	Favipiravir	~0.5^c^	[59•]
V791I	Remdesivir	500^b^	[58•]
C799F	Remdesivir	580^b^	[58•]
E802A	Remdesivir	2,700^a^	[57•]
E802D	Remdesivir	4,200^a^	[57•]
S861A	Remdesivir	*	[60]
S861G	Remdesivir	*	[61]
S861P	Remdesivir	*	[61]

a IC_50_ (half-maximal inhibitory concentration), indicates how much drug is necessary to inhibit the action of the process by half [44]

b EC_50_ (half-maximal effective concentration), indicates how much drug is necessary to induce a half-maximal response [62]

c ΔAffinity (relative binding affinity) representing the Boltzmann average of the relative affinities of the mutated proteins with respect to the wild-type protein. (higher ΔAffinity scores show unfavorable energetics which indicates potential resistance to Favipiravir binding) [59•]

* S861A, S861G, and S861P inhibit chain terminator mechanism, less efficiency to incorporate complementary UTP (Uridine Triphosphate) [60, 61]

** No mutational data for Molnupiravir impacting nsp12 is known

*** Various assays may be employed to obtain the findings; please refer to the respective publications for more information

## Data Availability

No datasets were generated or analysed during the current study.
